# Nuclear Phosphoinositides: Their Regulation and Roles in Nuclear Functions

**DOI:** 10.3390/ijms20122991

**Published:** 2019-06-19

**Authors:** R. Fiume, I. Faenza, B. Sheth, A. Poli, M.C. Vidalle, C. Mazzetti, S.H. Abdul, F. Campagnoli, M. Fabbrini, S.T. Kimber, G.A. Mariani, J. Xian, M.V. Marvi, S. Mongiorgi, Z. Shah, N. Divecha

**Affiliations:** 1 Department of Biomedical Sciences (DIBINEM), University of Bologna, Via Irnerio, 48, 40126 Bologna, Italy; irene.faenza2@unibo.it (I.F.); cristina.mazzetti13@gmail.com (C.M.); marco.fabbrini3@studio.unibo.it (M.F.); adalgisa.mariani@unibo.it (G.A.M.); jie.xian2@unibo.it (J.X.); mariavittoria.marvi@studio.unibo.it (M.V.M.); s.mongiorgi@unibo.it (S.M.); 2 Inositide Laboratory, School of Biological Sciences, Faculty of Environmental and Life Sciences, University of Southampton, Life Sciences Building 85, Highfield, Southampton SO17 1BJ, UK; B.Sheth@soton.ac.uk (B.S.); Magdalena.Castellano-Vidalle@soton.ac.uk (M.C.V.); S.Abdul-Hamid@soton.ac.uk (S.H.A.); f.campagnoli@soton.ac.uk (F.C.); S.T.Kimber@soton.ac.uk (S.T.K.); zash1000@yahoo.com (Z.S.); 3 Istituto FIRC di Oncologia Molecolare (IFOM), 20139 Milan, Italy; alessandro.poli@ifom.eu

**Keywords:** PtdIns(4,5)*P*_2_, PtdIns5*P*, phosphoinositides, epigenetic signalling, lipid kinase, nucleus, phospholipase C, demixing, liquid-liquid phase separation

## Abstract

Polyphosphoinositides (PPIns) are a family of seven lipid messengers that regulate a vast array of signalling pathways to control cell proliferation, migration, survival and differentiation. PPIns are differentially present in various sub-cellular compartments and, through the recruitment and regulation of specific proteins, are key regulators of compartment identity and function. Phosphoinositides and the enzymes that synthesise and degrade them are also present in the nuclear membrane and in nuclear membraneless compartments such as nuclear speckles. Here we discuss how PPIns in the nucleus are modulated in response to external cues and how they function to control downstream signalling. Finally we suggest a role for nuclear PPIns in liquid phase separations that are involved in the formation of membraneless compartments within the nucleus.

## 1. Introduction

**Figure 1:**
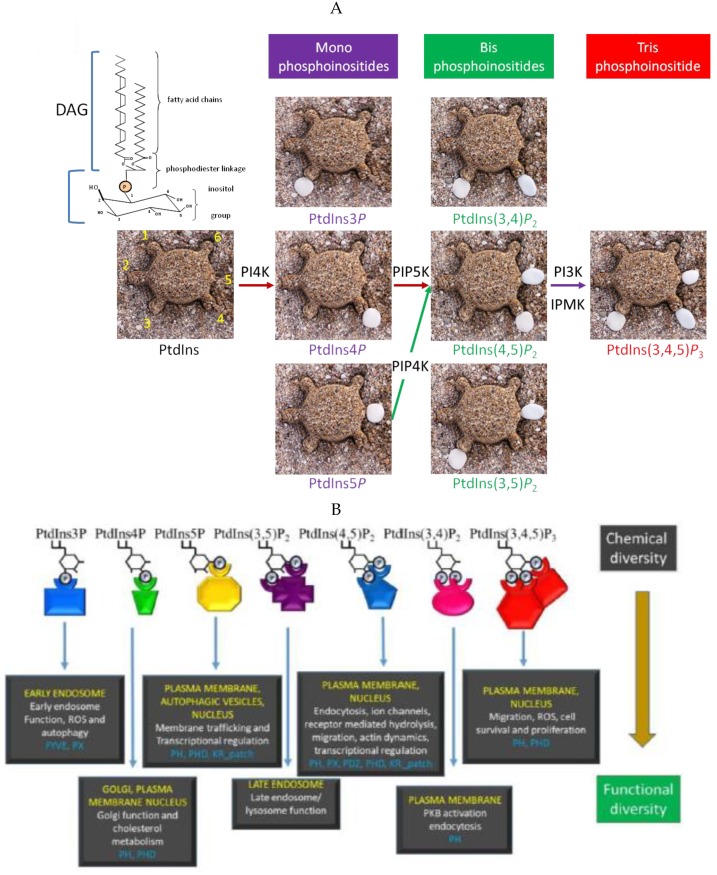
(**A**) The cartoon shows a representation of the parent phosphoinositide phosphatidylinositol, which can be phosphorylated on the 3, 4, 5 position of the head group to generate seven different PPIns. The 2-hydroxyl is shown in its axial conformation and in the turtles is represented by their head. The white pebbles represent phosphorylation sites. The red arrows show the canonical pathway for PtdIns(4,5)*P*_2_ synthesis, while the green arrow shows a minor pathway involving the PIP4K enzymes that phosphorylate PtdIns5*P* on the 4 position to generate PtdIns(4,5)*P*_2_. This pathway regulates both PtdIns5*P* and PtdIns(4,5)*P*_2_ levels. The purple arrow shows two pathways that generate PtdIns(3,4,5)*P*_3_ from the phosphorylation of PtdIns(4,5)*P*_2_. The IPMK pathway is not inhibited by classical PI3K inhibitors. (**B**) The seven PPIns recruit and regulate a panel of different downstream effectors to illicit differential outputs. In yellow are examples of subcellular compartments in which these lipids are known to function, while white refers to some of the functions they regulate. Examples of the types of domains each PPIns has been found to interact with are shown in blue. The chemical diversity of phosphoinositides through interaction with these binding proteins effectively transduces them into functionally diverse outputs.

Polyphosphoinositides (PPIns) are a family of phospholipids that are derived from phosphorylation of the parent molecule Phosphatidylinositol (PtdIns). PtdIns is composed of a diacylgylcerol (DAG) backbone coupled though a phosphodiester bond to a myo-inositol head group (Figure 1A). The inositol head group can be reversibly phosphorylated on the 3, 4 or 5 positions to generate seven unique PPIns. Given the hydrophobicity of the diacylglycerol backbone and the hydrophilicity of the phosphorylated inositol headgroup, PPIns function as an interactive boundary between the membrane and the cytoplasm. Recruitment of PPIns modulating enzymes to specific subcellular compartments generates unique PPIns profiles, which in turn localise target proteins to maintain compartment identity and function [1,2,3]. Target protein localisation occurs through specific lipid interaction motifs such as pleckstrin homology (PH) domains, Phox (PX) domains, FYVE domains or lysine arginine rich patches. PPIns regulate many cellular processes including membrane transport [2,4], ion channel function [5,6], cell adhesion, endo- and exo-cytosis [7], autophagy [8], transcription [1], RNA maturation [9] and cell survival [10] (Figure 1B). Importantly, many PPIns kinases and phosphatases are deregulated in various severe pathophysiological disorders such as cancer [11], myotubular myopathies [12] and Lowe syndrome [13]. In this review we will discuss the functional significance of phosphoinositides within the nucleus. We will discuss how they are regulated and example how they modulate downstream signalling to impact on nuclear functions. The presence of PPIns in the nucleus regulated by enzymes that are mutated in human diseases suggests that their dysregulation might be important in the development of human diseases.

## 2. PPIns in the Nucleus

**Figure 2:**
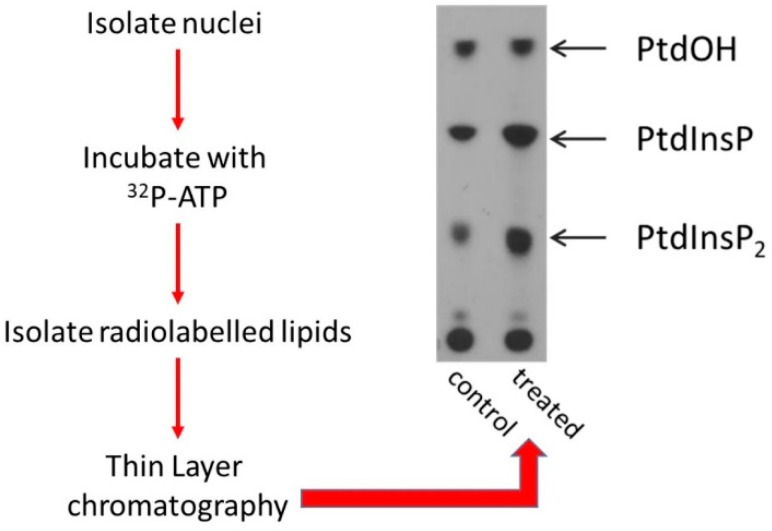
Nuclei from Murine erythroleukaemia cells (MEL) were isolated and incubated in the presence of ^32^Pγ-ATP for 5 minutes. The lipids were extracted and separated by thin layer chromatography. The control lane is nuclei from control murine erthroleukaemia (MEL) cells, while the treated lane shows nuclei isolated after differentiation of MEL cells into erythroblasts. Labelled PtdOH suggests that a nuclear DAG kinase has access to a pool of DAG, labelled PtdIns4*P* suggests that a nuclear PI-4-Kinase accesses a pool of PtdIns and labelled PtdIns(4,5)*P*_2_ suggests that a nuclear PIPK can access a pool of PtdIns4*P*. Note the increase in the labelling of nuclear PtdIns(4,5)*P*_2_. The right panel explains how label from ^32^Pγ-ATP is incorporated into the various lipids and defines substrates and enzymes.

Since the discovery that acetyl choline stimulation leads to changes in inositol lipid labelling, significant efforts have been invested to determine in which subcellular compartments inositol lipid turnover occurs. Early studies by Kai and Hawthorne [14] suggested that different compartments including the nucleus might contribute to changes in inositol lipid labelling. Given that the nuclear pool contributed only 10% of the total inositide pool that changed, contamination with other PPIns rich subcellular compartments could have accounted for this observation. A significant change in our understanding of nuclear PPIns occurred in 1983 when Smith and Wells [15,16,17] showed that phosphatidylinositol is not only present in the nucleus, but also that lipid kinases that generate Phosphatidylinositol(4,5)bisphosphate (PtdIns(4,5)*P*_2_) are also present. These observations came from a simple experiment using highly purified nuclear envelopes incubated with ^32^Pγ-ATP [17,18]. ^32^P-Labelling of Phosphatidylinositol-4-phosphate (PtdIns4*P*), PtdIns(4,5)*P*_2_ and phosphatidic acid show that the lipids PtdIns, PtdIns4*P* and DAG and enzymes that phosphorylate them are present in the nuclear envelope in close proximity to each other. A similar experiment to that of Smith and Wells is shown in Figure 2. Following radiolabelling of phosphoinositides in the “in nuclei” assay, addition of high concentrations of non-radioactive ATP or enzymatic depletion of the radiolabelled ATP led to a rapid decrease in labelled nuclear phosphoinositides. These data show that phosphatases and or phospholipases that degrade PPIns are also present in the nucleus [19]. PtdIns(4,5)*P*_2_ is the primary substrate for receptor stimulated phospholipase C generating two new second messengers, water soluble Ins(1,4,5)*P*_3_ and membrane bound DAG. The possibility that a similar pathway exists in the nucleus hints at novel mechanisms for phosphoinositide-mediated control of nuclear functions.

## 3. Phosphoinositides Are Present within the Nucleus and Not Just in the Nuclear Membrane

While studies carried out by Smith and Wells showed that nuclear PPIns and enzymes that can phosphorylate them are present in highly purified nuclear envelopes, nuclear fractionation studies aimed at removing the nuclear envelope also demonstrated that PPIns and PPIns-kinases are present inside the nucleus. Vann et al. [19] correlated nuclear envelope removal, assessed by electron microscopy, with the presence of PPIns and PPIns kinases. Washing highly purified rat liver nuclei with 0.04% triton X100 (TX-100) completely removed the nuclear membrane but surprisingly the mass levels of PtdIns(4,5)*P*_2_ only dropped to about 40%. Under similar conditions, “in nuclei” labelling with ^32^P-ATP showed significant labelling of PtdOH, PtdIns4*P* and PtdIns(4,5)*P*_2_. These data suggest that both phospholipids and lipid kinases are not only present in the nuclear envelope but are also present within the nucleus itself. Further studies showed that while PPIns lipid substrates are labile in TX-100, the PPIns kinases are tightly bound to the nuclear matrix. Subsequent fractionation studies showed that PtdIns-4-kinase (PI4K) is localised in the outer nuclear matrix while a PtdIns4*P*-5-kinase (PIP5K) was localised to the inner nuclear matrix [20]. These data suggest that PtdIns4*P*, the product of a nuclear PI4K might have its own roles within the nucleus. The generation of PtdIns4*P* in a location different from where it is required as a substrate for the synthesis of PtdIns(4,5)*P*_2_ might also suggest that lipid transport between these pools is important for nuclear PtdIns(4,5)*P*_2_ synthesis. Our unpublished studies show that there are at least two pools of PtdIns4*P* generated by two specific isoforms of PI4K. PI4Ks come in two flavours, one of which is inhibited by wortmannin [21] and another that is inhibited by adenosine [22]. Both types of PI4K are present in the nucleus and contribute to PtdIns4*P* synthesis, however, only the wortmannin-sensitive enzyme provides PtdIns4*P* that is further phosphorylated to PtdIns(4,5)*P*_2_.

## 4. The Intranuclear Location of PPIns in Membraneless Compartments: A Picture Paints A Thousand Words

**Figure 3:**
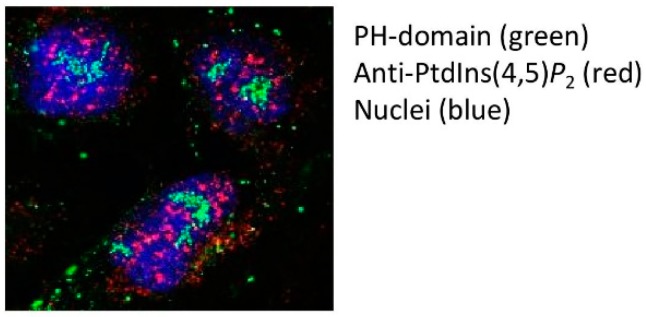
U2OS cells were fixed with 4% paraformaldehyde and permeabilised with PBS 0.1%TX-100 for 5 minutes. Nuclei were then incubated with bacterially expressed and purified GFP-PLCδ1 PH domain (green) and with the antibody (2C11) targeted to PtdIns(4,5)*P*_2_ (red staining). Nuclei were stained with DAPI (blue). The images from single channels were merged in Image J. Note the lack of overlap between the two types of PtdIns(4,5)*P*_2_ probes.

Even if the biochemical data are persuasive, nothing is more persuasive than a picture showing intranuclear localisation of phosphoinositides. Two tools have been used extensively to visualise PtdIns(4,5)*P*_2_ in the nucleus. The first relies on the specificity of the pleckstrin homology (PH) domain from Phospholipase C delta-1 (PLCδ1), while the second utilises a monoclonal IgM antibody raised to PtdIns(4,5)*P*_2_ [23]. Both reagents show high affinity and specificity for PtdIns(4,5)*P*_2_ over other phosphoinositides. In the case of the PH domain, mutation of specific residues can generate a control non-PtdIns(4,5)*P*_2_ interacting protein [24]. The simplest way of using both PtdIns(4,5)*P*_2_ detectors is to probe fixed and permeabilised cells and has been performed at both the light and electron microscope level [23,25,26,27,28]. To differing degrees both probes decorate the nuclear membrane, intranuclear speckles, or interchromatin granules, and to a lesser extent nucleoli. The mutant non-PtdIns(4,5)*P*_2_ interacting PH-domain control shows no staining within the nucleus and pre-treatment with neomycin, an aminoglycoside with high affinity for PtdIns(4,5)*P*_2_, also blocks nuclear decoration by both detectors. Although the nuclear staining pattern by both probes appear similar, our studies suggest that when the two probes are used to co-stain PtdIns(4,5)*P*_2_ they do not show strong overlap (Figure 3). These data have interesting ramifications as they suggest that the two probes recognise different pools of nuclear PtdIns(4,5)*P*_2_. The PH- probe would appear to be more specific to the nucleolar pool of PtdIns(4,5)*P*_2_ while the antibody strongly recognises nuclear speckles. 

## 5. What Is the Physiochemical Nature of PtdIns(4,5)*P*_2_ in Intranuclear Domains? 

PtdIns(4,5)*P*_2_ staining with the antibody strongly colocalises with SC35 (SRSF2) a nuclear protein that decorates nuclear speckles [23,28]. Nuclear speckles are membraneless compartments which begs the question as to the physico-chemical nature of PPIns in these structures. PPIns have hydrophobic tails that need to be shielded from the aqueous environment. This normally occurs as a consequence of insertion into a lipid bilayer. So how might PPIns be held within these membraneless compartments? Proteins with hydrophobic pockets could bind the PPIns tails and then present the head group for further phosphorylation, PLC mediated cleavage or target protein interaction. Indeed there is a family of PI transfer proteins (PITP) that carry out such functions and are found in the nucleus. Proteins of the TIPE2 homology family, such as TIPE3, interact with and present PPIns [29] and their artificial overexpression increases the total amount of PtdIns(4,5)*P*_2_ found in nuclear speckles. Similarly, overexpression of the MARCKS domain protein, another PtdIns(4,5)*P*_2_ interactor also increased nuclear PtdIns(4,5)*P*_2_ [30]. However, whether these are endogenous regulators of nuclear PtdIns(4,5)*P*_2_ is not known. Other studies suggest that a class of nuclear receptors, which include steroidogenic factor-1 (SF-1) and liver receptor homologue 1, can interact with PPIns lipid tails [31] and present the head group for phosphorylation [32,33]. Notably, these receptors regulate transcription of specific genes that are altered upon PPIns binding. Intranuclear PtdIns(4,5)*P*_2_ might also exist as a micellar structure discussed at the end of this review.

## 6. The Levels of Nuclear Phosphoinositides Change in Response to Various Extra and Intracellular Cues

In the cytoplasm different stimuli impact on the activity or localisation of phosphoinositide modulating enzymes to dynamically change PPIns profiles within subcellular compartments. For example receptor stimulation activates PI3-Kinase to generate a novel lipid surface rich in PtdIns(3,4,5)*P*_3_ that recruits proteins with PtdIns(3,4,5)*P*_3_ interaction domains, such as protein kinase B (PKB), to drive downstream signalling [34]. In 1987 a paper from the Cocco group [18] showed for the first time that nuclear PPIns changed after cells underwent differentiation (Figure 2). Further studies [35,36] showed that treatment of Swiss 3T3 cells with the growth factor IGF1, led to a decrease in nuclear PtdIns(4,5)*P*_2_ with a consequent increase in nuclear DAG. This was associated with translocation to the nucleus of Protein Kinase C (PKC), a known downstream target of DAG. These data are consistent with IGF1 inducing the activation of a nuclear PLC to hydrolyse PtdIns(4,5)*P*_2_. Subsequent studies have shown that nuclear phosphoinositides and the enzymes that modulate them form a signal transduction system that responds to growth factor treatment [35,36,37] cellular stressors [38], DNA damage [39], differentiation [18,40,41] and progression through the cell cycle [42]. In vivo changes in PPIns are also observed in liver nuclei in response to partial hepatectomy [43,44].

## 7. Changes in the Localisation or Activity of PPIns Modulating Enzymes Underlie Dynamic Changes in Nuclear PPIns

**Figure 4:**
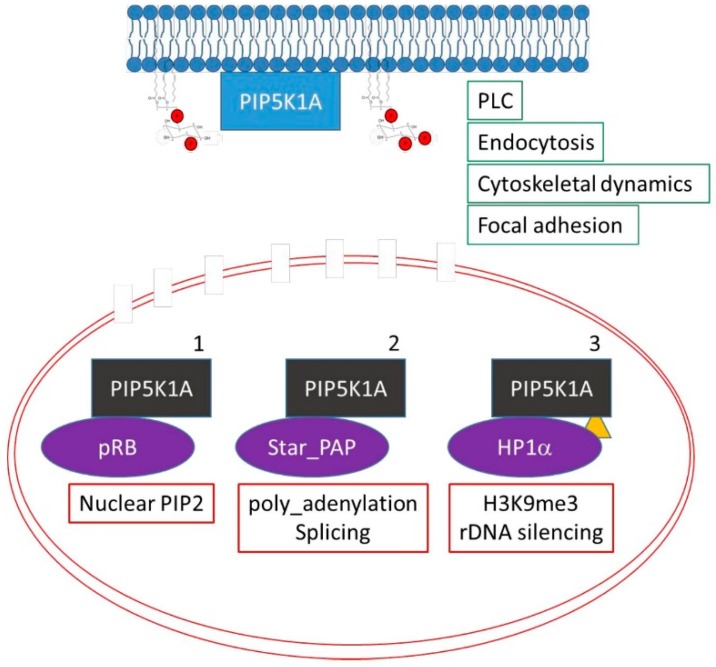
Three different mechanisms for localising PIP5K1A to the nucleus are shown. PIP5K1A has roles in the cytoplasm (blue) and can translocate into the nucleus (black). The change in the colour of PIP5K1A depicts changes in binding partners or in post translational modifications that enable nuclear localisation (the red lines depict the double nuclear membrane with some nuclear pores). The yellow triangle depicts sumoylation of PIP5K1A which has been shown to target PIP5K1A to the nucleus.

The previous studies illustrate how stimuli impact on the levels of nuclear phosphoinositides. However, most if not all of isoforms of PPIns modulating enzymes are present in multiple subcellular compartments and how they are regulated specifically within the nucleus is not clear. Here we highlight three pathways for nuclear specific regulation of PPIns modulators exemplified by the regulation of PIP5K1A (Figure 4). 

Firstly, the retinoblastoma protein (pRB), a tumour suppressor highly mutated in human tumors, is a nuclear scaffold protein that modulates transcription to control progression through the cell cycle, differentiation and DNA damage responses. Surprisingly, pRB is also a scaffold protein for lipid kinases such as PIP5K, PI3K and DAG kinase [45,46,47]. Lipids kinases interact with pRB through its pocket domain. In human tumours the pocket domain is often mutated, and these mutations attenuate interaction with lipid kinases. pRB regulates nuclear lipid synthesis, which play a role in pRB downstream signalling [45,46,47]. Given the prominent deregulation of pRB in tumours it is likely that nuclear lipid signalling plays an important role in tumour development. Secondly, Star-PAP, a non-canonical nuclear poly-A polymerase that modulates 3′mRNA maturation is strongly activated by nuclear PtdIns(4,5)*P*_2_ [9,48,49]. Star-PAP interacts with and localises PIP5K1A to the nucleus in order to increase the local production of PtdIns(4,5)*P*_2_. Thirdly, PIP5K1A is post-translationally modified by sumoylation [50,51] which controls its nuclear localisation and interaction with chromatin silencing complexes (Figure 4). 

Other studies on PIP4K2B have also illustrated how its activity and localisation is controlled in response to stimuli to impact on the levels of nuclear PPIns. In response to cellular stressors such as etoposide or oxidative damage or in response to differentiation there are increases in the levels of nuclear PtdIns5*P* [38,52,53,54]. Nuclear PtdIns5*P* can be removed by phosphorylation on the 4′ position to generate PtdIns(4,5)*P*_2_ by PIP4K2B [55]. Therefore PIP4K2B regulates two nuclear lipid messengers, PtdIns5*P* and PtdIns(4,5)*P*_2_. In response to either cellular stressors or myogenic differentiation the increase in nuclear PtdIns5*P* is associated with decreased nuclear PIP4K2B activity. However, this occurs in two distinct manners. Firstly in response to cell stress, the stress activated map kinase p38 phosphorylates PIP4K2B on serine 326 and threonine 322 which leads to a decrease in the activity of PIP4K2B [38]. In contrast, during differentiation of C2C12 myoblast cells into myotubes the decrease in nuclear PIP4K2B activity occurs as a direct consequence of PIP4K2B translocation out of the nucleus [56]. Other studies also showed that PIP4K2B localisation is under the control of extracellular stimulation [57]. PIP4K2B is also modified by ubiquitin by the nuclear E3 ligase SPOP [58] although the function of this modification is not clear. Similarly, the nuclear localisation and activity of PLCβ1 is also controlled by phosphorylation by MAPKinase [37]. Increased nuclear PLCβ1 activity controls cell proliferation and differentiation [59,60,61,62]. Recent unbiased studies have suggested that PLCβ1 can interact with a vast array of nuclear proteins, which are implicated in mRNA splicing and maturation, chromatin remodelling and in the regulation of apoptosis [63].

Clearly a thorough understanding of the interactions between phosphoinositide modulating enzymes and nuclear proteins will define how nuclear PPIns metabolising enzymes are controlled as well as which nuclear pathways are regulated directly by PPIns. 

## 8. Presentation of Lipids Is Important in Regulating Nuclear PPIns

A final example serves to illustrate how presentation of lipids impacts on the regulation of nuclear phosphoinositides. In response to DNA damage Kumar et al. demonstrated that p110β, which synthesises PtdIns(3,4,5)*P*_3_, is required for the recruitment of Nijmegan breakage syndrome protein 1 (NBS1) and the subsequent activation of the double strand break repair pathway. Surprisingly a scaffolding function of p110β rather than its activity is required in the damage response as illustrated by the lack of potency of PI3K inhibitors compared to RNAi mediated depletion of the protein. However, subsequent studies showed that nuclear PtdIns(3,4,5)*P*_3_ is important for activation of the double strand break repair pathway, but that its synthesis occurs predominantly by Inositol Polyphosphate Multikinase (IPMK). IPMK is an inositol kinase that generates highly phosphorylated inositols. However, IPMK can also generate PtdIns(3,4,5)*P*_3_ by phosphorylating PtdIns(4,5)*P*_2_ [64]. Classical PI3K inhibitors do not inhibit the lipid kinase activity of IPMK. What controls IPMK substrate specificity is not clear, but PtdIns(3,4,5)*P*_3_ synthesis is strongly dependent on presentation of PtdIns(4,5)*P*_2_. Within the nucleus an orphan steroid receptor called SF-1 binds the hydrophobic tail of PtdIns(4,5)*P*_2_ and presents the head group for phosphorylation. Surprisingly, PtdIns(4,5)*P*_2_ presented by SF1 is a very good substrate for IMPK to produce PtdIns(3,4,5)*P*_3_ but not for P110β [32]. In response to DNA damage SF1 presents PtdIns(4,5)*P*_2_ to IPMK which generates PtdIns(3,4,5)*P*_3_ at the double strand break [39]. IPMK and its activity in nuclear PtdIns(3,4,5)*P*_3_ formation has been implicated in human diseases [65]. 

## 9. How Do Phosphoinositides Impact on Nuclear Functions

The nucleus is a double membrane bounded organelle that houses the genetic makeup of a cell. Major functions within the nucleus include packing and unpacking DNA, DNA replication, DNA repair, RNA transcription and RNA splicing and export. DNA is wrapped around a histone octamer composed of two copies of four subunits of histone H2A, H2B, H3 and H4 to generate chromatin. Highly packed chromatin is repressive to most activities that require access to DNA and local unpacking of DNA is in part facilitated by post-translational modification of histone tails [66]. A plethora of histone tail modifications have been identified and include acetylation, methylation, ubiquitination and phosphorylation [66,67,68]. Acetylation of histone tails can physically disrupt local chromatin structure allowing access while other modifications such as methylation act as recruitment platforms for chromatin modifying complexes that in turn impact on chromatin structure. Chromatin Immuno-Precipitation and high throughput next generation sequencing (CHIP-seq) combined with mRNA analysis (RNA-seq or microarrays) show that specific histone tail modifications are associated with different gene transcriptional activation states [69]. For example, trimethylation of histone H3 lysine4 (H3K4me3) at gene promoters is associated with active transcription, while trimethylation at histone H3 lysine9 (H3K9me3) is associated with transcriptionally-inactive chromatin [70]. Notwithstanding that PPIns modulating enzymes may have scaffolding functions within the nucleus, we envisage two distinct ways in which PPIns might regulate nuclear functions. 

### 9.1. Nuclear PLC Pathway and Higher Phosphorylated Inositols

**Figure 5:**
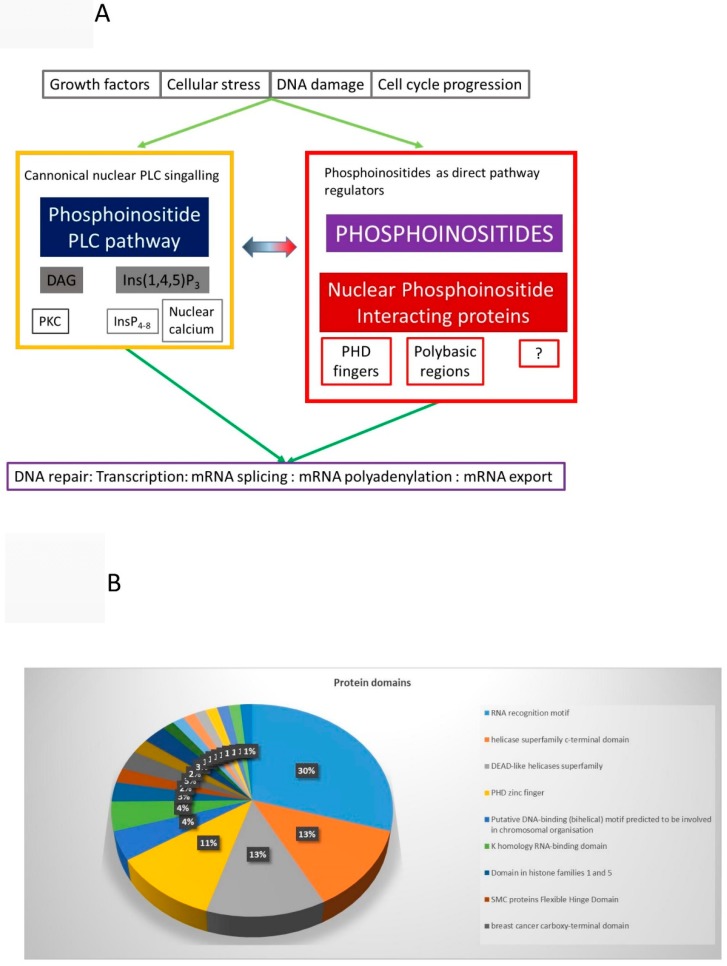

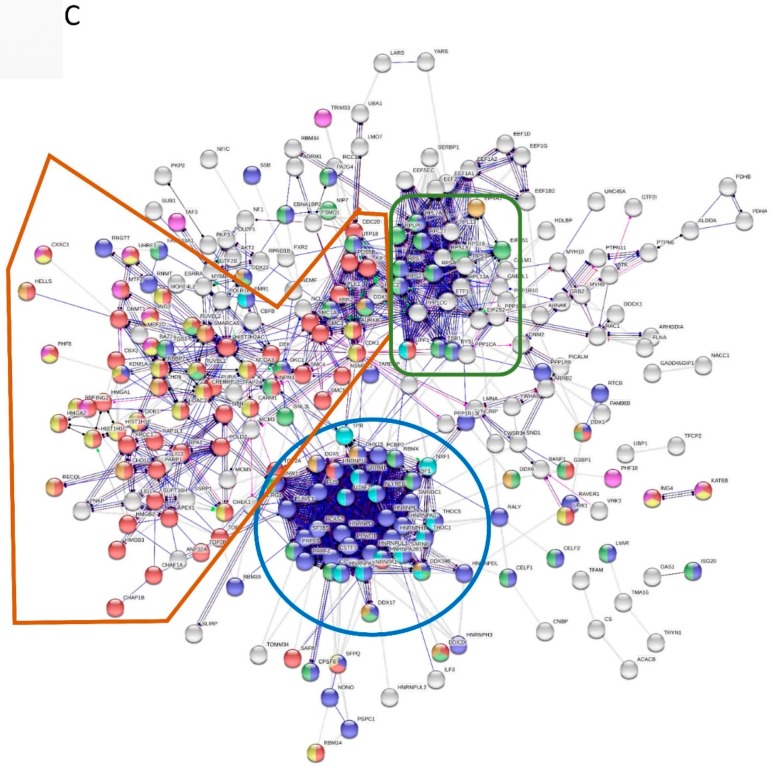
(**A**) Various types of extrinsic and intrinsic stimuli lead to the regulation of nuclear PPIns which impact on nuclear functions in two distinct ways. The first is through the nuclear PLC pathway that generates two second messengers, diacylgylcerol (DAG) and Ins(1,4,5)*P*_3_ which regulate Protein Kinase C (PKC) and nuclear calcium, respectively. In addition Ins(1,4,5)*P*_3_ can be further phosphorylated to higher phosphorylated inositol phosphates depicted by Ins*P*_4_-Ins*P*_8_. In the second box, changes in nuclear PPIns are transduced into functional outputs based on their ability to bind and to and regulate interacting proteins such as PHD finger containing proteins or those that contain polybasic regions. The ? depicts the presence of as yet unidentified nuclear PPIns binding proteins. (**B**) Smart domain enrichment in nuclear PPIns interacting proteins. (**C**) Proteins identified as interacting with PPIns or released from nuclei after incubation with neomycin were combined and protein IDs that were annotated as nuclear were extracted (349). Networks of protein were generated using STRING. Circle (nodes) are individual proteins and the lines that connect them (edges) represent modes of action and are based on interaction data extracted from text mining, experimental data and database searches. Unconnected nodes were eliminated from the figure for clarity. Nodes are coloured as below and can be multi-coloured.

With analogy to cytoplasmic PPIns signaling, activation of nuclear PLC hydrolyses PtdIns(4,5)*P*_2_ to generate DAG and Ins(1,4,5)*P*_3_ (Figure 5). Nuclear DAG recruits and activates PKC in the nucleus [35,36,43]. Nuclear substrates phosphorylated by PKC include lamins, topoisomerase1 and DNA polymerase [71,72,73,74,75,76]. Ins(1,4,5)*P*_3_ binds to Ins(1,4,5)P_3_ receptors present on the inner nuclear envelope [77] as well as on nuclear lipid bodies [78] to release calcium directly into the nucleus [77,79,80,81,82]. Increased nuclear calcium modulates calcium-calmodulin dependent protein kinases to regulate transcription [83]. Ins(1,4,5)*P*_3_ is also phosphorylated to highly phosphorylated inositols (Ins*P*_4__Ins*P*_8_), which in yeast regulate chromatin remodelling complexes [84,85,86], mRNA export [87], and telomere length [88,89]. Ins*P*_4_ in mammalian cells binds to and activates the HDAC3/NCOR2 complex to control protein acetylation [90,91]. Ins*P*_6_ binds to the Ku 70/80 subunit of the DNA dependent-protein kinase complex to regulate its activity and impact on non-homologous end joining during double strand break repair [92,93]. Ins*P*_6_ is also pyrophosphorylated to generate Ins*P*_7_ and Ins*P*_8_ [88]. Inositol hexakisphosphate kinase 1 and 2 (IP6K1 and 2) synthesise Ins*P*_7_ and IP6K1 interacts with KDM4C a H3K9me3 demethylase. Knockout of IP6K1 leads to reduced levels of H3K9me3, while its overexpression leads to increased H3K9me3 levels. These changes are dependent on the catalytic activity of IP6K1 implying that Ins*P*_7_ is a negative regulator of KDM4C [94,95]. Ins*P*_7_ might bind to KDM4C to negatively regulate its activity or could transfer a phosphate group directly to it [96]. H3K9 demethylases inhibitors are being developed as therapeutics for a number of cancers [97,98]. 


**Go-Process**

**Enrichment**

**FDR**

**Colour**
RNA processing80 of 8252.46e-34BlueChromosome organization80 of 9994.28e-29RedRibonucleoprotein complex biogenesis46 of 4091.79e-21GreenHistone modification28 of 3478.25e-10YellowmRNA transport15 of 1481.76e-06Dark GreenSmart Domain: DEAD-like helicases superfamily17 of 1091.22e-09Light BluePHD zinc finger15 of 916.98e-09Purple

The connecting lines (edges) are coloured as indicated below.


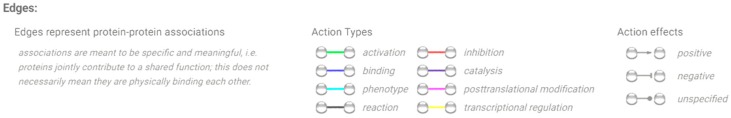


Within the figure subsets involved in RNA splicing and metabolism are circled in blue, while proteins involved in chromatin organisation in brown. Protein clustered in green are those involved in ribosome biogenesis.

### 9.2. Nuclear PPIns as Direct Regulators of Nuclear Function

PPIns also regulate downstream signalling by binding to specific proteins to regulate their activity, localisation or interactions with other proteins. It was therefore disappointing that most canonical phosphoinositide interaction motifs, such as the PH, FYVE or PX domains are not represented in bone fide nuclear proteins. A breakthrough came with the identification of the PHD finger of Inhibitor of Growth protein 2 (ING2) as a nuclear phosphoinositide receptor [38,54]. PHD fingers are cross braced zinc fingers, similar to the canonical PPIns binding FYVE domains, which are predominantly present in nuclear proteins. They function in protein-protein and protein-ligand interactions [99,100,101,102]. Notably, the PHD finger of ING2 interacts with both H3K4me3 and PtdIns5*P* [54,101,102]. In a small scale targeted screen, 16 out of 32 different PHD fingers were found to interact with PPIns implicating PPIns in the regulation of histone code reading, writing, and erasing and in chromatin remodelling and transcriptional activation/repression [56]. One of these, TAF3, is a core component of the basal transcriptional complex and PtdIns5*P* interaction regulates transcriptional output during myogenic differentiation [56]. Our studies outline important new roles for nuclear PPIns in epigenetic regulation. 

Other studies have shown that PtdIns(4,5)*P*_2_ interacts with and regulates histone H1-mediated RNA-polymerase inhibition [103], chromatin remodelling complexes [104,105], splicing factors, polyadenylation factors [9], mRNA export factors [106,107] and RNA polymerases [108,109,110]. ING2 regulation by PtdIns5*P* appears to regulate transcriptional output by p53 in response to stress. Recent studies have also shown that PtdIns(4,5)*P*_2_ synthesised by PIP5K1A controls the stability of p53 and in turn its transcriptional output [111]. To broaden the scope of phosphoinositide signalling in nuclear functions we carried out an unbiased proteomic identification of proteins that are released from nuclei after incubation with an aminoglycoside neomycin [112]. The logic behind this is that neomycin has very high affinity for PtdIns(4,5)*P*_2_ and is able to displace PtdIns(4,5)*P*_2_ interactors [112]. 349 proteins were found to be displaced by neomycin with a very small number of these containing canonical phosphoinositide interacting domains (PH, PHD). 168 proteins however, contained lysine/arginine rich patches previously identified as sequences in cytoskeletal proteins that mediate interaction with phosphoinositides. Two examples serve to illustrate the importance of these basic amino acid patches in the regulation of protein function. 

DNA topoisomerases I and IIα/β catalyse the passage of individual DNA strands (Topo I) or double strands (Topo II) through one another to impact on replication, transcription, recombination and chromosome segregation at mitosis. TopoIIα interacts with PtdIns(4,5)*P*_2_ through several basic patches located within its C-terminal domain. Addition of phosphoinositides strongly inhibited TopoIIα activity in vitro [112]. TopoIIα is also phosphorylated and activated by PKC [113,114] suggesting that nuclear PLC coordinates activation of TopoII by removing PtdIns(4,5)*P*_2_ and activating PKC.

UHRF1 (Ubiquitin like and ring finger containing protein) is also released from nuclei by treatment with neomycin. UHRF1 is a bridging protein that interacts with and regulates a family of DNA methyl-transferases (DNMT) [115,116]. DNMT activity is involved in DNA methylation maintenance during replication [117] and in the hypermethylation of CpG islands [118,119] in order to control tumour suppressor expression. Hypermethylated CpG islands are correlated with pathological grade, clinical stage and androgen-independence in prostate cancer. UHRF1 is upregulated in many human tumours including prostate [120], acts as an oncogene [121] and its suppression by RNAi attenuates cell growth [120]. UHRF1 contains two Tudor domains that mediate interaction with H3K9me3 and a PHD finger that interacts with non-methylated histone H3 [122,123]. These histone tail interactions are essential for proper UHRF1 function. The interaction with H3K9me3 is inhibited by a polybasic region in UHRF1 [124]. The polybasic region interacts with PtdIns5*P*, and this interaction modulates UHRF1 conformation enabling Tudor domain interaction with H3K9me3 [125]. 

To illustrate how nuclear PPIns interacting proteins might globally impact on nuclear physiology we combined data from a study that identified PPIns interacting proteins [126] with ours identifying proteins that are released by neomycin [112], together with our small scale study on PHD fingers [56]. Unique protein ID were identified and proteins classed as nuclear were extracted. This yielded 324 unique nuclear PPIns binding proteins (Table S1). This data set was then interrogated using STRING. Ontology analysis of this protein set showed, as expected, the term nuclear was the most significantly enriched cellular component. 502 GO-biological process terms were significantly enriched in this data set and include terms such “RNA metabolic processes”, “RNA splicing”, “RNA export”, “chromatin organisation” and “Histone modification” (Table S2). In addition several protein domains were highly enriched in this data set (Figure 5B) and include the RNA recognition motif (RRM), the PHD finger and the DEAD-like helicase superfamily (Table S3). A STRING interaction network was then generated (Figure 5C). The circles (nodes) represent individual protein IDs and the lines (edges) represent the action of the determined interaction, which might include binding, inhibition or activation and are based on experimental data, database analysis and text mining. At this level of complexity it is difficult to deconstruct the network, but what is clear is that certain types of nuclear functions appear to be highly targeted by PPIns binding. These include RNA maturation, ribosome biogenesis, chromatin organisation and histone modification. These data and others suggest that nuclear PPIns play a significant role in these functions through coordinated interaction with many different protein components [9,103,104,105,106,107]. 

## 10. Phosphoinositides as Potential Regulators of the Formation of Nuclear Membraneless Compartments

Electron and fluorescence light microscopy clearly show that PtdIns(4,5)*P*_2_ resides in nuclear speckles or interchromatin granules. Nuclear speckles were thought to be storage sites for pre-mRNA splicing factors [127] but are now known to contain over 500 proteins with the largest group being enriched in proteins involved in transcription [128] suggesting that they may couple transcription with mRNA splicing, maturation and export. The speckle interior lacks DNA and transcription occurs where DNA loops out and contacts the outer part of the speckle. As these loops can be derived from any chromosome, speckles might co-ordinately regulate the expression of genes required for specific functions acting as transcription factories [129]. Like many isolating domains in the nucleus, speckles are membraneless compartments that enable organisation, sequestration and concentration of components required for specific biochemical reactions [130,131,132]. These structures are thought to be formed by self-organising protein domains that undergo liquid-liquid phase separation (LLPS) similar to the oil water demixing that occurs after the two components are mixed. Liquid-liquid phase separation requires high concentrations of components coupled with multivalent interactions that lead to the formation of droplets with subsequent demixing of the components from the nucleosol. Critical components within nuclear speckles that enhance phase separation include RNA, RNA binding proteins (RNBP) and protein domains with intrinsically disordered stretches of aminoacids, which meditate promiscuous multivalent interactions [128,130]. Although, the physico-chemical organisation of nuclear PPIns in speckles is not clear they appear to be able to interact with many different RNA binding proteins [112] (Figure 5C). It is therefore tempting to speculate that micelles of PtdIns(4,5)*P*_2_, which spontaneously form, and by their nature are highly multivalent, might provide a seed for interaction and subsequent concentration of a heterogeneous population of RNA binding proteins. Together with the interaction between RNBPs and RNAs in the speckle, interaction with PPIns might provide additional stability and driving force for liquid phase separation and the generation of liquid like droplets. How PtdIns(4,5)*P*_2_ is targeted to the speckles is not clear, however, the stability of PtdIns(4,5)*P*_2_ within the speckle is dependent on RNA. Pre-treatment of nuclear speckles with RNAse, but not DNAse, leads to a loss of PtdIns(4,5)*P*_2_ within the speckle [23]. Finally, PPIns would also lead to concentration of many chromatin regulating proteins (Figure 5C) through phosphoinositide interacting domains that could then regulate transcription output. 

This proteolipid micellar structure could explain some of the experimentally observed characteristics of nuclear PPIns. It might provide a lipid surface that is detergent insensitive as observed during nuclear fractionation experiments [19]. In addition the multivalent nature of this micellar structure would allow immune-isolation of PtdIns(4,5)*P*_2_ via antibodies targeting the head group and still enable the isolation of proteins that bind to the micelle through head group interactions [23]. 

## 11. Conclusions

The regulation and function of nuclear phosphoinositides is likely to be important to determine how mutations in PPIns modulators impact on disease manifestation. While analysis of these mutations might initially appear to be explained by their cytoplasmic functions, the phenotypes observed in human disease, such as Lowe syndrome, are often complex and difficult to rationalise [133]. The presence of mutated PPIns modulators in the nucleus and downstream targets of nuclear PPIns signalling that impact on chromatin organisation, transcription, splicing, mRNA export and ribosome biogenesis and so can expand the mechanisms by which human disease maybe be manifested. 

**Figure 6:**
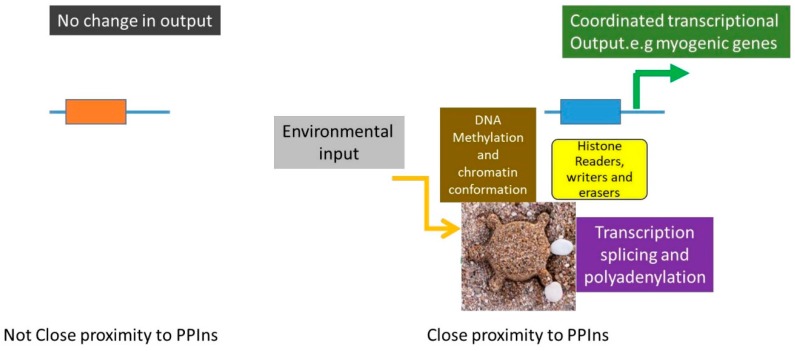
Depicts how changes in localised platforms of nuclear PPIns, such as at nuclear speckles, might impact on transcriptional output of genes that are in close proximity to the nuclear speckle through the recruitment of a group of proteins that modify DNA methylation, histone modification and regulate mRNA transcription, splicing and maturation. On the other hand genes that are not close to PPIns containing speckles would not be regulated by changes in nuclear PPIns. This would potentially explain selective gene regulation by PPIns binding proteins.

How exactly phosphoinositides are generated and maintained within the nucleus is still not clear and the current presumption is that PtdIns is transported into the nucleus and is phosphorylated to generate PtdIns(4,5)*P*_2_. Moreover, what exactly is the physicochemical environment of these lipids is not known. Possibilities include nuclear PPIns binding proteins that shield the hydrophobic tail, or as suggested here, self-assembling micelles and liquid-liquid phase separation that might enable the formation of stable structures that present PPIns in the nucleus. Given that nuclear PPIns are likely to be maintained within a specific environment how they regulate downstream proteins to impact on nuclear functions requires further studies. PPIns interaction might induce downstream target activation without continuous phosphoinositide interaction, enabling global target regulation within the nucleus. Alternatively and in keeping with the presence of PPIns in nuclear speckles, activation might be localised. In fact studies using mutations which attenuate PtdIns5*P* interaction in either ING2 or TAF3 show that PtdIns5*P* interaction regulates transcription of a subset of genes regulated by each of the wild type proteins [56,134]. How might this occur? The presence of PPIns in discrete nuclear domains such as speckles could act as platforms to recruit proteins which regulate chromatin organisation and transcriptional output. Genes that are looped out and are in close proximity to the speckle would then show regulation dependent on changes in PPIns whereas genes situated away from the speckle would not (Figure 6). Localised activation would explain the observed selective gene regulation.

Clearly, a more thorough understanding of how dynamic changes in nuclear PPIns impact on biological functions is necessary to reveal their exact role in human diseases. 

## Supplementary Materials

Supplementary materials can be found at https://www.mdpi.com/1422-0067/20/12/2991/s1.
